# Cholic acid therapy in Zellweger spectrum disorders

**DOI:** 10.1007/s10545-016-9962-9

**Published:** 2016-07-28

**Authors:** Kevin Berendse, Femke C. C. Klouwer, Bart G. P. Koot, Elles M. Kemper, Sacha Ferdinandusse, Kiran V. K. Koelfat, Martin Lenicek, Frank G. Schaap, Hans R. Waterham, Frédéric M. Vaz, Marc Engelen, Peter L. M. Jansen, Ronald J. A. Wanders, Bwee Tien Poll-The

**Affiliations:** 1 Department of Pediatric Neurology, Emma Children’s Hospital/Academic Medical Center, University of Amsterdam, Meibergdreef 9, 1105 AZ Amsterdam, The Netherlands; 2 Laboratory Genetic Metabolic Diseases, Academic Medical Center, University of Amsterdam, Amsterdam, The Netherlands; 3 Department of Pediatric Gastroenterology, Emma Children’s hospital/ Academic Medical Center, University of Amsterdam, Amsterdam, The Netherlands; 4 Department of Pharmacy, Academic Medical Center, University of Amsterdam, Amsterdam, The Netherlands; 5 Department of Surgery, Maastricht University, Amsterdam, The Netherlands; 6 Department of Medical Biochemistry and Laboratory Diagnostics, 1st Faculty of Medicine, Charles University in Prague, Prague, Czech Republic; 7 Department of Gastroenterology and Hepatology, Academic Medical Center, University of Amsterdam, Amsterdam, The Netherlands

## Abstract

**Introduction:**

Zellweger spectrum disorders (ZSDs) are characterized by a failure in peroxisome formation, caused by autosomal recessive mutations in different *PEX* genes. At least some of the progressive and irreversible clinical abnormalities in patients with a ZSD, particularly liver dysfunction, are likely caused by the accumulation of toxic bile acid intermediates. We investigated whether cholic acid supplementation can suppress bile acid synthesis, reduce accumulation of toxic bile acid intermediates and improve liver function in these patients.

**Methods:**

An open label, pretest-posttest design study was conducted including 19 patients with a ZSD. Participants were followed longitudinally during a period of 2.5 years prior to the start of the intervention. Subsequently, all patients received oral cholic acid and were followed during 9 months of treatment. Bile acids, peroxisomal metabolites, liver function and liver stiffness were measured at baseline and 4, 12 and 36 weeks after start of cholic acid treatment.

**Results:**

During cholic acid treatment, bile acid synthesis decreased in the majority of patients. Reduced levels of bile acid intermediates were found in plasma and excretion of bile acid intermediates in urine was diminished. In patients with advanced liver disease (*n* = 4), cholic acid treatment resulted in increased levels of plasma transaminases, bilirubin and cholic acid with only a minor reduction in bile acid intermediates.

**Conclusions:**

Oral cholic acid therapy can be used in the majority of patients with a ZSD, leading to at least partial suppression of bile acid synthesis. However, caution is needed in patients with advanced liver disease due to possible hepatotoxic effects.

**Electronic supplementary material:**

The online version of this article (doi:10.1007/s10545-016-9962-9) contains supplementary material, which is available to authorized users.

## Introduction

The Zellweger spectrum disorders (ZSDs) constitute the main group within the peroxisome biogenesis disorders and are characterized by a deficiency of functional peroxisomes due to mutations in different *PEX* genes. The clinical spectrum is very heterogeneous, but key symptoms are liver disease, visual and auditory impairment and developmental delay (Gould et al 2001). In patients with a more severe phenotype, advanced fibrosis and biliary cirrhosis is already present soon after birth (Wanders and Ferdinandusse 2012). Clinically, hepatosplenomegaly, elevated plasma transaminases, cholestasis and steatorrhea are often present. Due to the deficiency of functional peroxisomes, several metabolite abnormalities are usually found in ZSD patients. Typically, patients accumulate C_27_-bile acid intermediates, very long-chain fatty acids, phytanic, pristanic and pipecolic acid in plasma and have low levels of plasmalogens in erythrocytes (Wanders and Waterham 2006). The bile acid abnormalities in ZSDs are thought to contribute to overall disease pathogenesis, but especially to liver disease pathology (Wanders and Ferdinandusse 2012). Moreover, it is hypothesized that the C_27_-bile acid intermediates cross the blood–brain barrier and cause central nerve system damage (Klouwer et al 2015).

Bile acids are water-soluble derivatives of cholesterol which play an important role in a range of metabolic processes, such as cholesterol catabolism and dietary lipid absorption (including fat-soluble vitamins). In addition, they are signalling molecules in multiple metabolic pathways (Hofmann 2007). The primary bile acids cholic acid (CA) and chenodeoxycholic acid (CDCA) are synthesized in the liver from cholesterol via multiple enzymatic steps in different subcellular compartments (i.e. mitochondrion, cytosol, endoplasmic reticulum and peroxisome) (Russell 2003; Zollner et al 2006). The final step in primary bile acid synthesis takes place in the peroxisomes: the C_27_-bile acids 3α,7α-dihydroxycholestanoic acid (DHCA) and 3α,7α,12α-trihydroxycholestanoic acid (THCA) undergo one cycle of peroxisomal β-oxidation to yield the primary C_24_-bile acids CDCA and CA. In ZSD patients, accumulation of these C_27_-bile acid intermediates occurs together with inadequate concentrations of C_24_-bile acids in patients with a severe phenotype (Ferdinandusse and Houten 2006; Van Eldere et al 1987). Accumulating bile acids are hepatotoxic (Fischer et al 1996). Moreover, in vitro studies have shown that C_27_-bile acids are more cytotoxic than C_24_-bile acids (Ferdinandusse et al 2009). Primary bile acids are normally conjugated with either taurine or glycine to enhance their aqueous solubility via the peroxisomal enzyme bile acid-coenzyme A: amino acid N-acyl transferase (BAAT) (Pircher et al 2003). ZSD patients have decreased conjugating capacity due to the deficiency of peroxisomal BAAT (Solaas et al 2000). In humans, C_27_-bile acid intermediates are less well conjugated in comparison to C_24_-bile acids, are poorly secreted and fail to generate sufficient bile flow, promoting cholestasis. After secretion they are less able to form mixed micelles in the intestinal lumen, leading to malabsorption of fat and fat-soluble vitamins (Van Eldere et al 1987; Stieger et al 1997).

Bile acids regulate their own biosynthesis via a negative feedback loop. They have been identified as endogenous ligands of the farnesoid X receptor (FXR) (for a review see Schaap et al 2014). Activation of FXR in the liver results in reduced gene expression of cholesterol 7α-hydroxylase (CYP7A1), the rate-limiting enzyme in de novo synthesis of bile acids (Brendel et al 2002; Goodwin et al 2000; Kerr et al 2002). In addition, FXR is activated in the intestine after bile acid reabsorption, which induces production of fibroblast growth factor 19 (FGF19). This endocrine factor acts on the liver to downregulate *CYP7A1*. The majority of bile acids are reabsorbed in the ileum and return to the liver via portal blood (i.e. enterohepatic circulation), whereas only a small amount is excreted in the feces. To maintain a constant bile acid pool, the loss is compensated by de novo synthesis in the liver (Chiang 2009). In ZSDs, bile acid synthesis is upregulated as a likely consequence of decreased levels of C_24_-bile acids and attendant impairment of negative feedback regulation via FXR/FGF19 signaling. This leads to increased production and subsequent accumulation of C_27_- bile acid intermediates (Keane et al 2007).

Currently, no curative therapy for patients with ZSDs exists (Klouwer et al 2015). Supplementation of CA is hypothesized to be a potential therapy for patients with a ZSD (Setchell et al 1992), as CA represses the first step in biosynthesis of bile acids, possibly leading to reduced levels of bile acid intermediates. In addition, CA therapy is likely to restore the reduced CA levels, thereby improving bile flow and increasing solubilisation of dietary fats and fat-soluble vitamins. CA therapy was recently approved in the United States by the Food and Drug Administration (FDA) as a treatment for patients with ZSDs. Until now, the effect of primary bile acid supplementation has been reported in three ZSD patients with inconsistent results (Setchell et al 1992; Maeda et al 2002), and its effect has never been studied systematically in a large cohort. In this study, we investigated the effect of 9 months of oral CA therapy in 19 patients with a ZSD.

## Methods

### Study design

The study was approved by the Ethics Committee of the Academic Medical Center (AMC), Amsterdam, The Netherlands and took place between 2011 and 2014 (trial registry: www.isrctn.com/ISRCTN96480891). Individual written informed consent was obtained from the patients and/or the patients’ parents. Due to the wide spectrum of disease severity and orphan character of the disease, a pretest-posttest design was used. The pre-treatment phase was defined as 2.5 years prior to start of the treatment, during this period patients were under standard care and follow-up. Patients were examined during the treatment phase at three study visits; 4, 12 and 36 weeks after start of CA treatment. During these visits, a physical examination and liver stiffness measurement were performed. Furthermore, blood and urine were collected for biochemical analyses.

Primary study objectives were to determine: 1) the degree of suppression of bile acid synthesis (as defined by the change in plasma levels of the C_27_-bile acid intermediates DHCA and THCA, FGF19 and 7α-hydroxy-4-cholesten-3-one [C4]) and urinary occurrence of bile acid intermediates, and 2) the change in plasma C_24_-bile acid levels. Concentration of C4, a stable bile acid intermediate, is a serum marker for CYP7A1 activity (Axelson et al 1988). Secondary study objectives comprised: changes in liver elasticity, fat-soluble vitamin levels, liver protein synthesis, markers of peroxisomal function and monitoring of possible side effects of CA treatment including: change in plasma transaminases (aspartate transaminase [AST], alanine transaminase [ALT]) and conjugated bilirubin.

### Patients

Inclusion criteria comprised: genetically confirmed ZSD and at least one of the following hallmarks: elevated transaminases, growth retardation or neurological symptoms. Exclusion criterion was a life expectancy of less than 1 year (i.e. patients with the classic Zellweger syndrome phenotype or patients receiving palliative care), but since none of the patients in our cohort met this exclusion criterion at the time of enrolment, no patients were excluded. Nineteen patients were recruited for this study. All patients attended the outpatient clinic of the AMC for study visits, and were examined by a pediatric neurologist. Vitamin supplementation remained unchanged in all patients throughout the study.

### CA

Active Pharmaceutical Ingredient (API) of CA was provided by Asklepion Pharmaceuticals (since 2015 Retrophin, Inc., NY, United States) and analysed amongst other things for identity, purity and related substances by a HPLC method. Capsules were developed and manufactured according to the guidelines of Good Manufacturing Practice commissioned by the AMC resulting in two dosage forms; yellow 50 mg capsules and opaque 250 mg capsules. CA capsules of 50 and/or 250 mg were administered twice a day orally during or before meals with a total dosage of 15 mg/kg/day. The dosage was increased to 20 mg/kg/day in case C_27_-bile acid intermediates DHCA and/or THCA were still detectable in plasma. Effect of dose escalation was checked after 4 weeks. In case of clinical side effects, particularly diarrhoea, vomiting or biochemical side effects defined as a twofold increase in plasma transaminases or conjugated bilirubin, the dosage was reduced to 10 mg/kg/day. No placebo was used.

### Biochemical analysis, liver stiffness measurements and physical examination

Plasma and urinary bile acids (Bootsma et al 1999; Blau et al 2003), plasma very long-chain fatty acids, phytanic acid, pristanic acid (Vreken et al 1998), pipecolic acid (Rashed et al 2001) and plasmalogens in erythrocytes (Dacremont and Vincent 1995) were measured at the Laboratory Genetic Metabolic Diseases in the AMC. The urinary bile acids analysed comprise, among others, primary bile acids (conjugates), bile alcohols and C_27_-bile acid intermediates as described earlier (Ferdinandusse and Houten 2006). The detection limit of bile acid intermediates in this assay is 0.05 μmol/L. Plasma FGF19 was determined as described previously (Schreuder et al 2010) and plasma C4 was measured by LC-MS based on the original method of (Lenicek et al 2008), with a detection limit of 1 ng/mL. For detailed LC-MS conditions see supplementary methods. Standard diagnostic assays were used to measure low-density lipoprotein, high-density lipoprotein and total cholesterol, albumin and coagulation factors (i.e. prothrombin, partial thromboplastin time, factor V and VII). Liver stiffness analyses were performed by a single trained observer using transient elastography Fibroscan® according to the standard manufacturer instructions (Echosens, Paris, France). This ultrasound-based method measures propagation speed of a shear wave in liver tissue which is well validated to measure liver stiffness, and is considered the most accurate non-invasive method for diagnosis of liver cirrhosis (de Lédinghen et al 2007; Chang et al 2016). Since Fibroscan® values have not been validated against liver histology for ZSD patients, the METAVIR fibrosis scale for chronic cholestatic liver disease was used. A Fibroscan® value ≥15.5 kPa was defined as severe fibrosis or cirrhosis. All patients underwent standard physical and neurological examinations at each study visit. Weight was measured with a calibrated balance, standard deviation (SD) scores relative to the general population were calculated following current Dutch standards (Talma et al 2010).

### Statistical analysis

A Wilcoxon matched-pairs signed-rank sum test was used to evaluate effects (baseline vs. the individual follow-up time points) of CA supplementation, using the IBM Statistical package for the Social Sciences (SPSS) software version 22 (IBM, USA). A p-value of <0.05 was considered as statistically significant.

## Results

Patient characteristics are presented in Table 1. No patients were lost during follow-up. All patients, except patient 16, received CA supplementation for 9 months with a starting dose of 15 mg/kg/day. CA supplementation was generally well tolerated and no patients discontinued their medication. In three patients (1, 18, 19) CA was administrated via a gastrostomy. CA dose was increased to 20 mg/kg/day in five patients at different time points due to persistently elevated levels of C_27_-bile acid intermediates in plasma (patient 12–15, 19). The dose was decreased to 10 mg/kg/day in four patients due to diarrhoea (patient 2,3), a twofold increase in plasma transaminases (patient 1) or a rise in conjugated bilirubin (patient 16). This latter patient (16) dropped out due to persistently elevated conjugated bilirubin levels after dose reduction, and was excluded from the study analysis after 36 weeks of treatment (Table 2). After cessation of CA supplementation in this patient, plasma levels of conjugated bilirubin, transaminases and CA returned to pre-treatment levels (data not shown).

**Table 1:** Clinical and genetic characteristics of 19 ZSD patients treated with CA

Mutation							
Patient #	Gender	Age at start of treatment in years	Allele 1	Allele 1	CA dose after 36 weeks of treatment in mg/kg/day	Vitamin supplementation	Other
1	F	7	PEX1 c.1007T>C, c.1663T>C	PEX1 c.2845C>T	10	K	PEG, epilepsy
2	M	23	PEX1 c.2528G>A	PEX1 c.2528G>A	10	D, E, K	
3	F	15	PEX1 c.2528G>A	PEX1 c.2528G>A	10	D, E, K	
4^a^	F	35	PEX1 c.2528G>A	PEX1 c.2528G>A	15	A, E, K	
5^a^	M	30	PEX1 c.2528G>A	PEX1 c.2528G>A	15	A, E, K	
6^b^	F	17	PEX1 c.2528G>A	PEX1 c.2528G>A	15	D, E, K	
7	F	17	PEX1 c.2528G>A	PEX1 c.2528G>A	15	D, E, K	
8	M	8	PEX1 c.2528G>A	PEX1 c.2528G>A	15	A, D, E, K	
9	F	18	PEX6 c.1801C>T	PEX1 c.1992G>C	15	A, D, E, K	
10	F	20	PEX1 c.1777G>A	PEX1 c.2071 + 1G>T	15	E, K	
11^c^	M	9	PEX1 c.2528G>A	PEX1 c.2528G>A	15	A, D, K	
12	M	16	PEX26 c.292C>T	PEX26 c.292C>T	20	D, E, K	
13	F	8	PEX10 c.1A>G	PEX1 c.199C>T	20	E, K	
14^b^	M	12	PEX1 c.2528G>A	PEX1 c.2528G>A	20	A, D, E, K	
15^c^	F	2	PEX1 c.2528G>A	PEX1 c.2528G>A	20	D, E, K	
16	M	4	PEX1 c.2528G>A	PEX1 c.2614C>T	dropped out	A,K	
17	F	7	PEX1 c.2528G>A	PEX1 c.2528G>A	15	E,K	
18	F	8	PEX1 c.2528G>A	PEX1 c.2528G>A	15	A, D, E, K	PEG, epilepsy
19	F	10	PEX1 c.2097lnsT	PEX1 c.2528G>A	20	A, E, K	PEG, epilepsy

Abbreviation: *CA* cholic acid, *PEG* Percutaneous endoscopic gastrostomy. ^a^ sibs, ^b^ sibs, ^c^ sibs

**Table 2:** Biochemical analyses in plasma and urine from ZSD patients treated with CA (group 1: patients 1–15, group 2: patients 16–19)

CA treatment	0 weeks						4 weeks			12 weeks						36 weeks					
Patient #	CA	DHCA	THCA	FGF19	C4	Urine#	CA	DHCA	THCA	CA	DHCA	THCA	FGF19	C4	Urine#	CA	DHCA	THCA	FGF19	C4	Urine#
1	0.5	0.2	0.6	0.999	30.6	present	3.0	<0.05	0.1	**3.5**	**0.3**	**0.1**	**0.545**	**<1.0**	**nd**	**2.2**	**0.3**	**0.1**	**0.444**	**4.3**	**nd**
2	0.5	<0.05	<0.05	0.413	24.3	nd	6.2	<0.05	<0.05	**1.0**	**<0.05**	**<0.05**	**0.193**	**11.6**	**nd**	**2.6**	**<0.05**	**<0.05**	**0.507**	**12.7**	**nd**
3	2.8	0.2	0.4	0.242	12.3	nd	**10.0**	**0.1**	**0.2**	**2.2**	**<0.05**	**<0.05**			**nd**	**1.6**	**0.1**	**<0.05**	**0.286**	**7.2**	**nd**
4	0.8	1.6	0.9	0.055	19.1	nd	7.7	0.4	<0.05	3.7	0.5	0.1	0.647	1.0	nd	2.7	<0.05	0.1	1.166	<1.0	nd
5	0.1	0.3	0.1	0.186	27.4	nd	3.4	<0.05	<0.05	0.4	<0.05	<0.05	0.601	<1.9	nd	0.2	<0.05	<0.05	0.387	4.4	nd
6	0.3	<0.05	<0.05	0.134	23.5	present	3.5	<0.05	<0.05	4.0	<0.05	<0.05	1.284	1.0	nd	0.8	<0.05	<0.05	0.356	<1.0	nd
7	0.6	<0.05	<0.05	0.449	28.7	nd	2.4	<0.05	<0.05	1.0	<0.05	<0.05	0.248	10.0	nd	1.0	<0.05	<0.05	0.390	17.5	nd
8	1.0	0.2	0.4	0.475	18.9	nd	2.4	<0.05	<0.05	9.1	<0.05	0.3	0.799	1.6	nd	2.6	0.2	0.1	0.885	<1.0	nd
9	0.4	<0.05	0.1	0.100	12.1	nd	8.3	<0.05	<0.05	5.5	<0.05	<0.05	0.914	1.2	nd	1.9	<0.05	0.1	0.453	1.4	nd
10	0.1	<0.05	<0.05			nd	1.3	<0.05	<0.05	1.1	<0.05	<0.05	0.847	11.7	nd	1.3	<0.05	<0.05	0.208	7.7	nd
11	1.0	0.2	<0.05	0.406	29.2	nd	1.6	<0.05	<0.05	4.0	<0.05	0.1	0.686	14.2	nd	6.5	<0.05	0.1	0.328	15.2	nd
12	1.4	2.8	7.2	0.181	11.3	present	15.1	1.0	2.4	*21.6*	*1.3*	*1.4*	*0.319*	*1.7*	*nd*	*39.9*	*1.3*	*2.5*	*0.573*	*1.2*	*nd*
13	0.6	0.5	2.0	0.142	<1.0	nd	7.2	0.4	0.1	*10.1*	*0.4*	*<0.05*	*0.328*	*<1.0*	*nd*	*13.3*	*0.3*	*0.4*	*0.353*	*<1.0*	*nd*
14	0.5	4.1	0.4	0.114	34.3	present	11.3	0.5	0.2	*15.1*	*<0.05*	*<0.05*	*0.817*	*<1.0*	*nd*	*9.9*	*<0.05*	*0.1*	*0.422*	*<1.0*	*nd*
15	3.3	4.4	1.7	0.177	8.4	nd	12.2	1.1	0.7	9.6	1.2	0.6	0.276	2.5	nd	*13.0*	*0.8*	*0.8*	*0.240*	*3.6*	*nd*
16	3.3	6.0	10.6	0.359	2.4	present	174.8	3.3	10.0	**69.4**	**3.2**	**16.7**	**0.516**	**<1.0**	**present**				excluded		
17	47.4	6.0	18.5	0.254	1.7	present	116.6	1.0	1.5	123.2	1.6	1.7	0.483	<1.0	nd	251.8	1.5	3.7	0.815	<1.0	nd
18	11.0	10.8	32.4	0.423	1.8	no urine	92.4	3.8	8.9	175.8	3.9	9.0	1.053	<1.0	no urine	154.2	4.9	10.1	1.083	<1.0	no urine
19	2.4	15.5	12.0	0.094	1.1	present	60.0	9.3	6.2	*20.6*	*8.3*	*3.6*	*0.460*	*<1.0*	*present*	*100.2*	*10.5*	*11.5*	*0.374*	*<1.0*	*nd*
Median	0.8	0.3	0.4	0.2	15.6		7.7	0.1	0.1	5.5	0.0	0.1	0.6	1.1		2.7	0.1	0.1	0.4	1.3	

*Abbreviation*: *CA* cholic acid, *DHCA* dihydroxycholestanoic acid, *THCA* trihydroxycholestanoic acid, *FGF19* fibroblast growth factor 19, *C4* 7alpha-hydroxy-4-cholesten-3-one, *nd* not detected. # Urinary bile acids comprises C_27_-bile acid intermediates, derivatives and bile alcohols. Plasma bile acids in μmol/L, FGF19 and C4 in μg/L; 10 mg/kg/day cholic acid doses are depicted in bold, 20 mg/kg/day cholic acid doses are depicted in italic. Due to problems with blood withdrawal or hemolysis of the samples, some data points are missing. Note that urinary bile acid were only measured qualitatively

Note: Significance of bold and italic - effect of different doses of cholic acid supplementation on different biochemical parameters

### Bile acid analysis

**Fig. 1:**
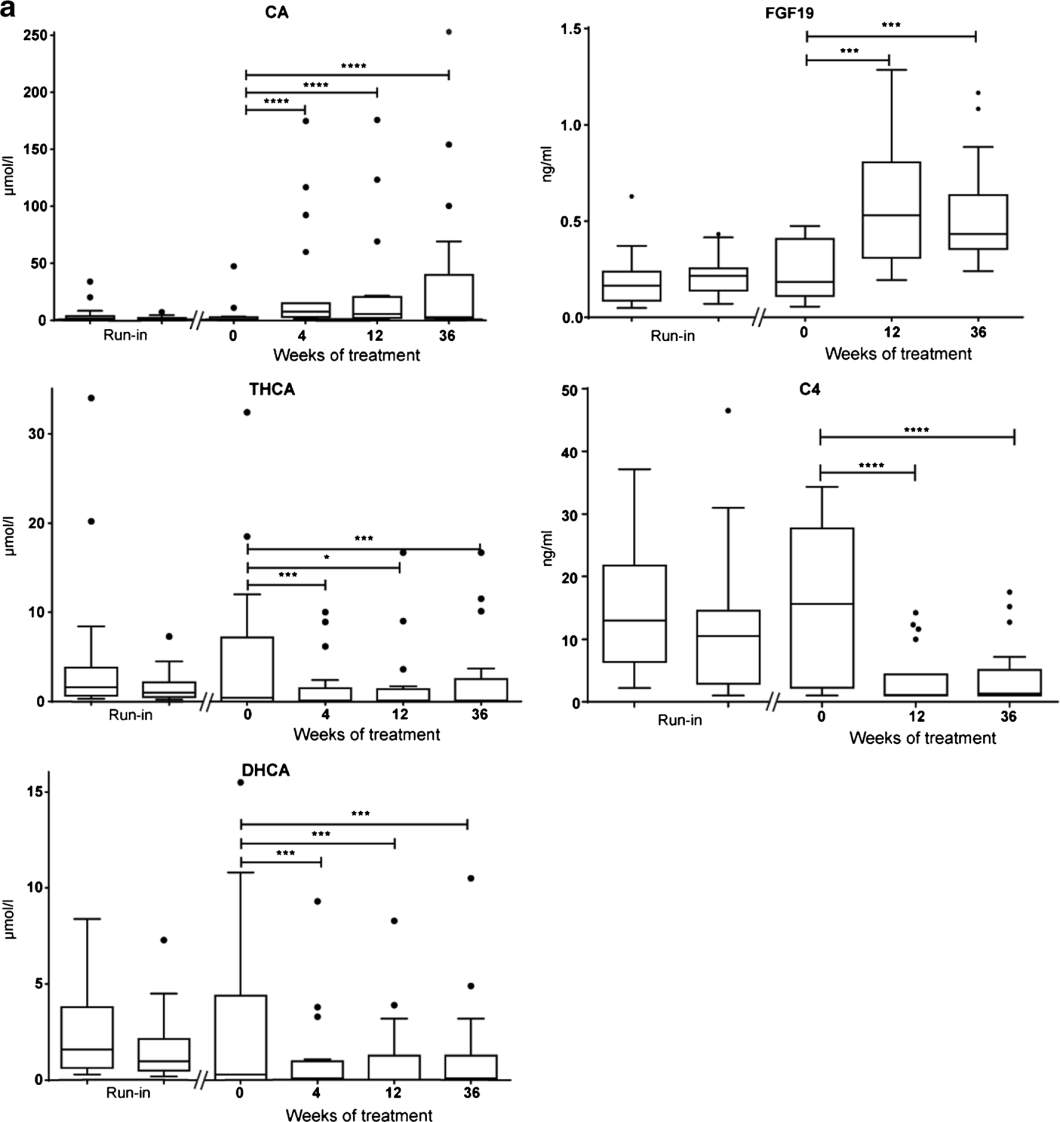

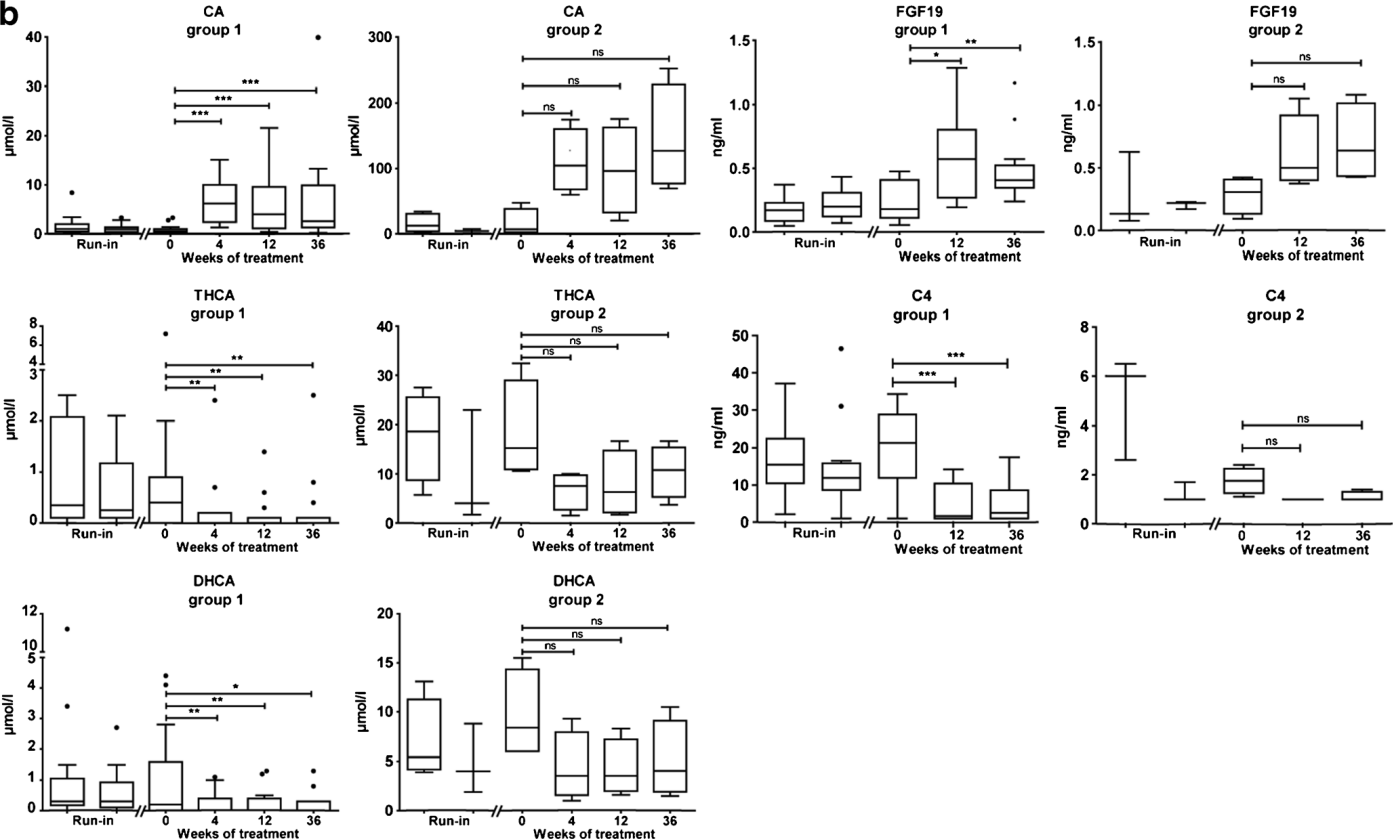
**a** Tuckey boxplots showing the effect of oral cholic acid (CA) therapy on plasma CA concentrations, 3α,7α,12α-trihydroxycholestanoic acid (THCA), 3α,7α-dihydroxycholestanoic acid (DHCA), fibroblast growth factor 19 (FGF19) and 7α-hydroxy-4-cholesten-3-one (C4) in the entire cohort of patients with a ZSD (*n* = 19). Run-in was defined as 2.5 and 2 years prior to treatment. The reference range of CA is 0.1–4.7 μmol/L and THCA <0.05–0.1 μmol/L. Levels of DHCA are undetectable (<0.05 μmol/L) in controls. No reference range for FGF19 and C4 in children exists. Statistical analyses were performed with a Wilcoxon matched-pairs signed-rank sum test. *P < 0.05; ***P < 0.005; ****P < 0.001. *Abbreviation: ns* not significant. **b** Tuckey boxplots showing the effect of oral cholic acid (CA) therapy on plasma CA concentrations, 3α,7α,12α-trihydroxycholestanoic acid (THCA), 3α,7α-dihydroxycholestanoic acid (DHCA), fibroblast growth factor 19 (FGF19) and 7α-hydroxy-4-cholesten-3-one (C4) in subgroups defined by baseline liver stiffness values. Run-in was defined as 2.5 and 2 years prior to treatment. The reference range of CA is 0.1–4.7 μmol/L and THCA <0.05–0.1 μmol/L. Levels of DHCA are undetectable (<0.05 μmol/L) in controls. No reference range for FGF19 and C4 in children exists. Statistical analyses were performed with a Wilcoxon matched-pairs signed-rank sum test. *P < 0.05; **P < 0.01; ***P < 0.005. *Abbreviation*: *ns* not significant

Detailed biochemical data of the individual patients are presented in Table 2, with the bile acids depicted as the sum of unconjugated and conjugated bile acids. As illustrated in Fig. 1a, plasma CA levels increased significantly during CA supplementation. Both DHCA and THCA levels decreased significantly after 4, 12 and 36 weeks of CA treatment compared to baseline. Plasma levels of FGF19, a negative regulator of *CYP7A1* expression, and C4, a marker for CYP7A1 enzyme activity, were respectively increased and decreased after 12 and 36 weeks of CA treatment. As seen in Table 2, in some patients (16–19) a strong rise in CA (100–250 μmol/l) was observed in plasma upon supplementation with CA. In addition, levels of DHCA and THCA in these patients remained markedly increased during CA treatment. Levels of the C_29_-dicarboxylic acid remained stable during treatment in all patients (data not shown).

The four patients with high plasma CA levels and persistently elevated levels of C_27_-bile acid intermediates all had Fibroscan® values ≥15.5 kPa, whereas all other patients had values below 15.5 kPa. Therefore, we divided the group into two subgroups based on the degree of liver stiffness determined by Fibroscan® analysis prior to therapy (group1 Fibroscan <15.5 [*n* = 15] and group 2 Fibroscan ≥15.5 kPa [*n* = 4]) and performed post hoc analysis. As illustrated in Fig. 1b, the increase in plasma CA, as well as the decrease in DHCA and THCA following CA supplementation was significant in group 1 but not in group 2. Also the therapy-induced changes in FGF19 and C4 levels were only significant in group 1. It should be noted that the levels of C4 in group 2 were already remarkably low at baseline. The high levels of total CA in plasma, especially in group 2, consisted mainly of conjugated CA.

Urinary excretion of C_27_-bile acid intermediates, derivatives and bile alcohols was seen at baseline in patients 6, 12, 14, 16–17 and 19. Urine collection was not possible in patient 18. After 36 weeks of treatment, these latter metabolites were undetectable in urine of all patients studied.

### Liver function tests

**Fig. 2:**
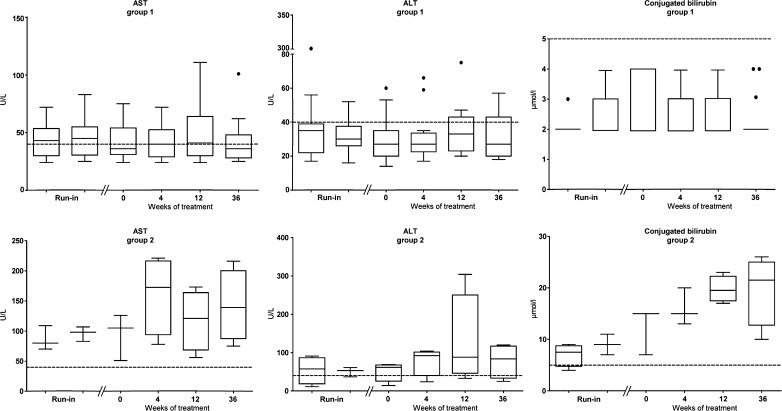
Tuckey boxplots showing the effect of oral cholic acid (CA) therapy on plasma levels of aspartate transaminase (AST), alanine transaminase (ALT) and conjugated bilirubin in subgroups defined by baseline liver stiffness values. Run-in was defined as 2.5 and 2 years prior to treatment. The reference range (depicted as a dashed line in each boxplot) of AST is 0–45 U/L, of ALT 0–40 U/L and of conjugated bilirubin 0–5 μmol/L. Statistical analyses were performed with a Wilcoxon matched-pairs signed-rank sum test. CA supplementation had no significant effects on these parameters at any of the studied time points after start of therapy

Median AST, ALT and conjugated bilirubin levels were normal at the start of therapy and remained unaltered during CA supplementation. In addition, individual levels in patients of group 1 remained stable (Fig. 2). Although the plasma transaminases and/or conjugated bilirubin levels were increased before treatment in the majority of patients in group 2, all these parameters further increased, albeit not significantly, during CA supplementation (Fig. 2).

### Fibroscan measurements

Fibroscan® liver stiffness values did not change significantly in either group during the 36 weeks of CA treatment. The median value prior to start of therapy was 7.9 kPa (range 4.8–39.7) compared to median 6.6 kPa (range 3.25–44.4) after 9 months of treatment (Supplementary Table 1).

### Secondary measures

No changes from baseline were observed in the concentrations of fat-soluble vitamins (A, E and D), coagulation parameters, cholesterol and albumin levels after 36 weeks of CA treatment. The levels of other peroxisomal parameters, such as very long-chain fatty acids, pristanic, phytanic and pipecolic acid in plasma, and plasmalogens in erythrocytes, remained unchanged. No difference in the median SD score for weight was observed (data not shown).

## Discussion

CA, marketed as Cholbam® in the United States, was recently approved by the FDA for treatment of patients with a ZSD under a ‘rare pediatric disease priority review voucher’ (FDA 2015). Until now, CA therapy has not been studied in a large cohort of ZSD patients. In this study, we report the results of 9 months CA supplementation in a group of 19 patients with a ZSD, and show that CA can be used in a subgroup of ZSD patients without severe liver disease. It should be noted, however, that in patients with advanced liver disease oral CA treatment might be harmful, as our study showed increased levels of plasma transaminases, conjugated bilirubin and markedly increased CA levels in plasma without a notable effect on bile acid synthesis in these patients.

Because the subgroup of ZSD patients with advanced liver disease seemed to react differently to CA treatment, we divided the cohort into two groups based on the degree of liver stiffness, measured with the Fibroscan®. Patients in group 1 (patients 1–15) had values <15.5 kPa and patients in group 2 (patients 16–19) scored ≥15. 5kPa (15.5 kPa is known to correlate with bridging fibrosis and cirrhosis). Overall, patients in group 1 responded well to the CA treatment, with a significant decline in C_27_-bile acid intermediates levels in plasma and urine and suppressed levels of C4 in plasma, albeit in some patients DHCA and THCA remained detectable in plasma (patient 12, 13, 15). In four out of 13 patients (31 %) with elevated levels of DHCA/THCA at baseline, these bile acid intermediates were undetectable after 9 months of CA treatment. In the patients with undetectable bile acid intermediates in plasma at baseline, suppression of bile acid synthesis could only be judged on the basis of C4 levels, which showed increased suppression during the course of the intervention in two out of four patients (patient 2, 6).

Patients with cirrhosis (i.e. group 2), showed a marked increase in CA levels in plasma upon treatment (up to 250 μmol/L in patient 17) and only a small reduction in the plasma levels of the C_27_-bile acid intermediates. In addition, liver enzymes and conjugated bilirubin levels increased, suggesting liver damage. It should be noted that all patients in this group already had low levels of C4 at baseline, indicating reduced bile acid synthesis. This is probably related to the high levels of bile acid intermediates DHCA and THCA, that are known to act as low affinity ligands of FXR, causing downregulation of *CYP7A1* and accordingly reduced bile acid synthesis (Nishimaki-Mogami et al 2004). It should be realized that under these conditions Na+ taurocholate cotransporting polypeptide (NTCP), the bile acid transporter mediating bile acid uptake in the liver, is also downregulated. This contributes to the lack of response to CA in cirrhotic patients. Because levels of FGF19 were normal in these patients, bile acid intermediates are likely to activate FXR directly in the liver and not via the usual route, activation of FGF19 in the terminal part of the ileum. This may be related to the low affinity of DHCA and THCA. Concentrations reached in the ileum may be too low to activate FXR (Nishimaki-Mogami et al 2004). Upon CA supplementation, C4 levels further decreased to or below 1.0 ng/mL, paralleled by a significant increase in FGF19 concentrations. This additional suppression of bile acid synthesis likely involving FGF19 signalling, is reflected in the observed reduction in C_27_-bile acid intermediates, albeit minor. Despite the suppressed bile acid biosynthesis in these patients, as deduced from the low levels of C4, bile acid intermediates DHCA and THCA were still detectable in plasma. This is probably due to the liver cirrhosis in these patients, leading to an inadequate bile flow, leakage of bile and reversed transport of bile acids and intermediates from liver to plasma by the cholestasis-induced membrane transporters MRP3, MRP4 and OSTα/β in the hepatocyte basolateral plasma membrane (Halilbasic et al 2013). This hypothesis is confirmed by the marked increase of mainly conjugated bile acids in plasma, as bile acids are conjugated in the liver. Furthermore, it is noteworthy that patients with low levels of bile acid intermediates had high levels of C4 and low levels of FGF19 in plasma in contrast to those with high levels of DHCA and THCA (Table 2).

Treatment with a combination of CA and CDCA was previously reported to lower bile acid intermediates in urine and plasma in a single ZSD patient with a severe phenotype. Additionally, levels of liver enzymes in plasma declined, growth rate improved and the degree of steatorrhea reduced (Setchell et al 1992). Another two ZSD patients were treated with CDCA and/or ursodeoxycholic acid (UDCA) with different results on biochemical outcome. CDCA treatment alone resulted in decreased levels of THCA and DHCA in urine and plasma, but with an increase in the total serum bilirubin level. Similar results were found with UDCA treatment, albeit without increase in plasma transaminases and bilirubin. The second patient was treated with UDCA combined with CDCA, leading to decreased bile acid intermediates in plasma. Short single treatment with CDCA resulted in an increase of ALT, which recovered after the initiation of UDCA (Maeda et al 2002). Furthermore, it has been reported that liver enzymes levels in plasma improved in patients with a defect in bile acid synthesis (Gonzales et al 2009). In our study, we also observed decreased levels of DHCA and THCA in urine and plasma, but no positive effect on liver enzymes was observed. It should be noted that some patients, especially those in group 1, already had normal levels of AST, ALT and conjugated bilirubin at baseline. In patients with an increased AST and/or ALT at baseline (patient 1, 11, 13–19), these levels remained unaltered or increased after treatment with CA, particularly in those with advanced liver disease.

Limitations of our study are that the degree of steatorrhea and the effect of CA therapy on this parameter was not measured, the small size of the cohort, especially the number of patients in group 2, and the lack of a placebo arm in this trial. Despite the small number of patients in group 2, the correlation between the advanced liver disease and increase in plasma ALT, AST and conjugated bilirubin upon cholic acid treatment in these patients seems to be clearly present. This is supported by the finding that the levels of conjugated bilirubin, ALT and AST returned to baseline after cessation of cholic acid supplementation in one of these four patients (patient 16). Furthermore, Fibroscan® liver stiffness measurement is an accurate method to diagnose liver cirrhosis, which we used to define our patient groups, but is probably less useful to discriminate between various stages of liver fibrosis and therefore to assess therapeutic effect of CA (Chang et al 2016).

In conclusion, our results indicate that oral CA supplementation can be given to ZSD patients without advanced liver disease, leading to at least partial suppression of bile acid synthesis in the majority of patients. Because our study shows that CA can be potentially harmful for patients with advanced liver disease, we discourage to treat this subgroup of ZSD patients with CA. The presence of advanced liver disease, such as cirrhosis, can be determined using Fibroscan® measurements, but it is also possible to diagnose patients using standard ultrasound examination or by performing a liver biopsy. In patients with a milder liver phenotype, the treatment period of 9 months was too short to be able to conclude whether CA had an effect on clinical progression in patients with a ZSD, since this is a slowly progressive disorder. In this study we chose for CA rather than UDCA treatment, because CA is a better FXR ligand than UDCA and CA. CA supplementation may help to ameliorate the nutritional deficiencies of ZSD patients by its detergent effects in the intestine. However, our study shows that CA treatment of ZSD patients is complex and effects may not only be beneficial. In cholestatic patients toxic effects of CA treatment prevail over possible beneficial effects. In these patients UDCA treatment may be a better choice. Additional long-term studies are necessary to assess the benefits of CA therapy on relevant clinical endpoints (i.e. effect on growth, liver disease, neurological deterioration) and long-term safety in ZSD patients.

## Electronic supplementary material

Below is the link to the electronic supplementary material.

ESM 1 (TIF 4878 kb)

ESM 2 (DOCX 16 kb)

## Abbreviations

ALT: Alanine transaminase
AST: Aspartate transaminase
CA: Cholic acid
CDCA: Chenodeoxycholic acid
CYP7A1: Cholesterol 7α-hydroxylase
C4: 7α-hydroxy-4-cholesten-3-one
DHCA: 3α,7α-dihydroxycholestanoic acid
FDA: Food and drug administration
FGF19: Fibroblast growth factor 19
FXR: Farnesoid X receptor
SD: Standard deviation
THCA: 3α,7α,12α-trihydroxycholestanoic acid
UDCA: Ursodeoxycholic acid
ZSD: Zellweger spectrum disorder
