# Supplementary figures and images for: Cholic acid therapy in Zellweger spectrum disorders
Source: J Inherit Metab Dis. 2016 Jul 28;39(6):859–68. doi: 10.1007/s10545-016-9962-9 (PMC5065608; doi:10.1007/s10545-016-9962-9)

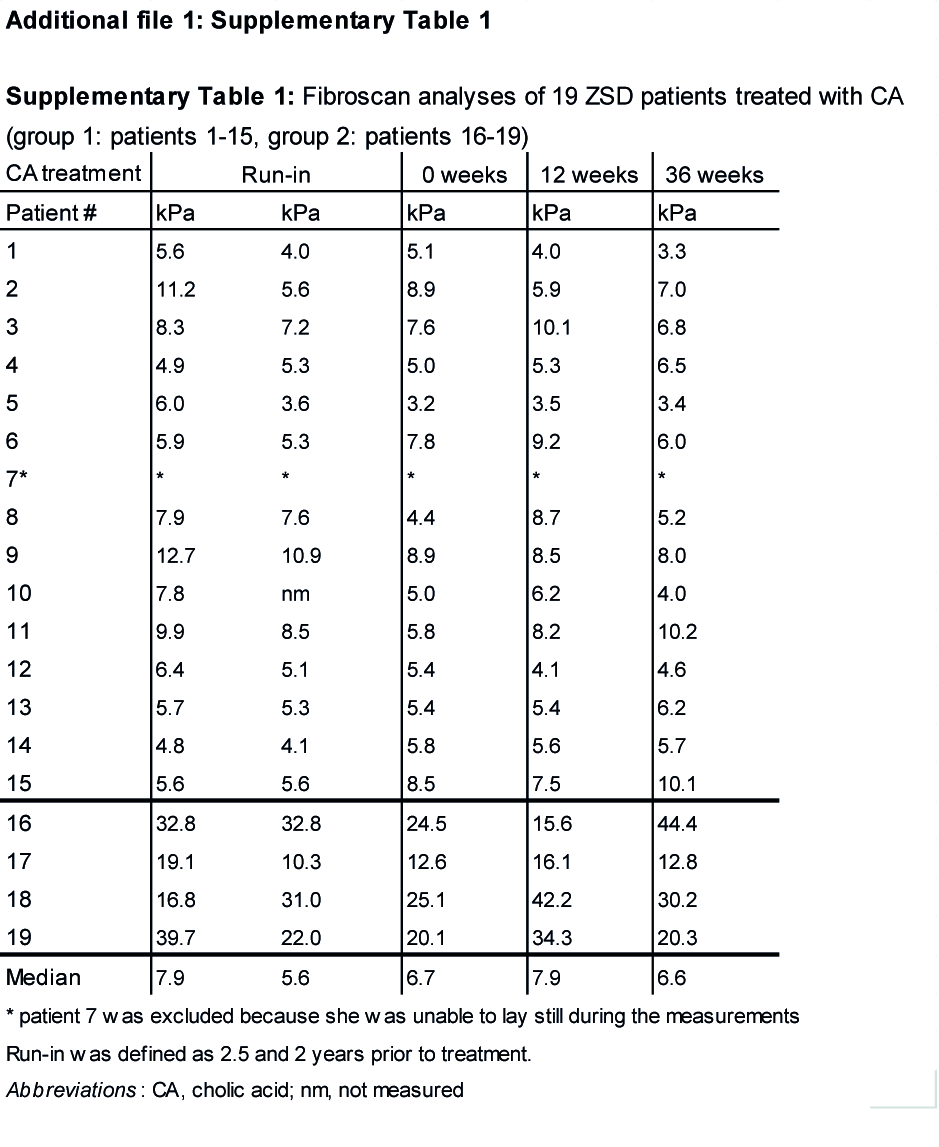

Supplement: Supplementary file 1 — (TIF 4878 kb) [file 10545_2016_9962_MOESM1_ESM.tif]
