# Supplementary material for: Cholic acid therapy in Zellweger spectrum disorders
Source: J Inherit Metab Dis. 2016 Jul 28;39(6):859–68. doi: 10.1007/s10545-016-9962-9 (PMC5065608; doi:10.1007/s10545-016-9962-9)
Supplement: Supplementary file 2 — (DOCX 16 kb) [file 10545_2016_9962_MOESM2_ESM.docx]

Supplementary material to:

**Cholic acid therapy in Zellweger spectrum disorders**

Kevin Berendse^1, 2^ ¶, Femke C. C. Klouwer^1, 2^ ¶, Bart G.P. Koot^3^ ¶, Elles M. Kemper^4^, Sacha Ferdinandusse^2^, Kiran V.K. Koelfat^5^, Martin Lenicek^6,^ Frank G. Schaap^5^, Hans R. Waterham^2^, Frédéric M. Vaz^2^, Marc Engelen^1^, Peter L.M. Jansen^7^, Ronald J.A. Wanders^2^, Bwee Tien Poll-The^1^ *

¶ Equal contributors

^1^ Department of Pediatric Neurology, Emma Children’s Hospital/Academic Medical Center , University of Amsterdam, The Netherlands. ^2^ Laboratory Genetic Metabolic Diseases, Academic Medical Center, University of Amsterdam, The Netherlands. ^3^ Department of Pediatric Gastroenterology, Emma Children’s hospital/ Academic Medical Center, University of Amsterdam, The Netherlands. ^4^ Department of Pharmacy, Academic Medical Center, University of Amsterdam, The Netherlands. ^5^  Department of Surgery, Maastricht University, The Netherlands. ^6^ Department of Medical Biochemistry and Laboratory Diagnostics, 1^st^ Faculty of Medicine, Charles University in Prague, Czech Republic. ^7^ Department of Gastroenterology and Hepatology, Academic Medical Center, University of Amsterdam, The Netherlands.

*Corresponding author: Bwee Tien Poll-The, Department of Pediatric Neurology, Emma Children’s Hospital, Academic Medical Center, University of Amsterdam, Meibergdreef 9, 1105 AZ Amsterdam, The Netherlands. Phone: +31-20-5667508. Email: b.t.pollthe@amc.uva.nl

**LC-MS measurement of 7α-hydroxy-4-cholesten-3-one (C4)**

Material

LC-MS grade methanol (Biosolve BV, The Netherlands), LC-MS grade ammonium acetate (Sigma-Aldrich, USA)

Methods

One hundred microliters of serum and 2 ng of internal standard (7α-Hydroxy-4-cholesten-3-one d7, Santa Cruz Biotechnology, USA) in 40 µL of methanol were mixed and extracted as previously reported (Lenicek et al 2008). Purified sample was dissolved in 50 µL of 75% methanol, 15 µL were injected on HPLC system (Dionex Ultimate 3000, Dionex Softron GmbH, Germany) equipped with Hypersil GOLD column (150x2.1 mm, 3 µm, Thermo Scientific, USA) and SecurityGuard column (Phenomenex, USA). Sample was eluted with methanol:water:ammonium acetate (flow rate 0.3 mL/min) at 40°C. While ammonium acetate concentration was kept at 0.1% (w/v) at all times, methanol concentrations (v/v) were as follows: 1.-8. min 82 %-90 %; 8.-10. min 90 %; 10.-12. min 99 %; 12.-17. min 82 %. Triple quadrupole mass spectrometer (TSQ Quantum Access Max with H-ESI II probe, Thermo Fisher Scientific, Inc., USA) operating in SIM mode served as detector. Transitions used for monitoring of C4 and internal standard were: m/z 401.4 → 177.3, 401.4 → 383.6 and 408.4 → 184.3, 408.4 → 390.6, respectively.

References

Lenicek M, Juklova M, Zelenka J et al. Improved HPLC Analysis of Serum 7{alpha}-Hydroxycholest-4-en-3-one, a Marker of Bile Acid Malabsorption. *Clin Chem* 2008;54:1087-88.
